# Relationships Between Immune Landscapes, Genetic Subtypes and Responses to Immunotherapy in Colorectal Cancer

**DOI:** 10.3389/fimmu.2020.00369

**Published:** 2020-03-06

**Authors:** Emilie Picard, Chris P. Verschoor, Grace W. Ma, Graham Pawelec

**Affiliations:** ^1^Health Sciences North Research Institute, Sudbury, ON, Canada; ^2^Department of Surgery, Health Sciences North, Sudbury, ON, Canada; ^3^Department of Immunology, University of Tübingen, Tübingen, Germany

**Keywords:** colorectal cancer, microsatellite instability, immunotherapy, checkpoint blockade, immunoscore, tumor-infiltrating lymphocytes, neoantigens, vaccination

## Abstract

Colorectal cancer (CRC) is highly heterogeneous at the genetic and molecular level, which has major repercussions on the efficacy of immunotherapy. A small subset of CRCs exhibit microsatellite instability (MSI), a molecular indicator of defective DNA mismatch repair (MMR), but the majority are microsatellite-stable (MSS). The high tumor mutational burden (TMB) and neoantigen load in MSI tumors favors the infiltration of immune effector cells, and antitumor immune responses within these tumors are strong relative to their MSS counterparts. MSI has emerged as a major predictive marker for the efficacy of immune checkpoint blockade over the last few years and nivolumab or pembrolizumab targeting PD-1 has been approved for patients with MSI refractory or metastatic CRC. However, some MSS tumors show DNA polymerase epsilon (POLE) mutations that also confer a very high TMB and may also be heavily infiltrated by immune cells making them amenable to respond to immune checkpoint inhibitors (ICI). In this review we discuss the role of the different immune landscapes in CRC and their relationships with defined CRC genetic subtypes. We discuss potential reasons why immune checkpoint blockade has met with limited success for the majority of CRC patients, despite the finding that immune cell infiltration of primary non-metastatic tumors is a strong predictive, and prognostic factor for relapse and survival. We then consider in which ways CRC cells develop mechanisms to resist ICI. Finally, we address the latest advances in CRC vaccination and how a personalized neoantigen vaccine strategy might overcome the resistance of MSI and MSS tumors in patients for whom immune checkpoint blockade is not a treatment option.

## Introduction

Colorectal cancer (CRC) is the third most often-diagnosed cancer in both men and women, with more than 1.8 million new cases worldwide in 2018 (1). CRC development is generally slow, asymptomatic and follows a multistep course. The tumor arises from a benign polyp and is driven by the accumulation of genetic mutations and epigenetic changes. These events induce histological and morphological changes leading to a carcinoma, which can spread to lymph nodes and adjacent or distant organs in the most advanced stage of its development (2). CRC patients’ clinical outcome is closely related to the tumor and nodal stage. While the 5-year survival rates are around 90% and 71% for patients with localized and regional tumors, respectively, this rate decreases dramatically to 13–14% in the setting of distant metastasis (3). However, discrepancies in terms of prognosis were observed between patients with the same disease stage and were associated with different genetic mutations, highlighting the molecular heterogeneity of CRC (4). A major genetic modification in CRC relies on the impairment of DNA mismatch repair (MMR) activity leading to a microsatellite instability (MSI) phenotype in 15% of tumors, different from the majority of microsatellite stable (MSS) tumors without such impairment which represent 85% of CRC cases (5). Recently, numerous studies have investigated the immune cells in the microenvironment of CRC. First considered poorly immunogenic, it is now established that CRCs display a heterogeneous immune landscape, according to their microsatellite status and other factors. While most CRC patients have MSS tumors with a poor immune cell infiltration, a subset of patients with tumors of the MSI phenotype is characterized by tumors enriched with immune cells and expressing neoantigens that activate antitumor immune responses (6). It has been shown that the infiltration of specific subsets of functional immune cells within these tumors is associated with an improved prognosis and a low risk of recurrence after surgery in patients with stage I, II, or III CRC (7). The presence of immune cells in this subset of MSI CRC supports the notion that a treatment based on immunotherapies should provide clinical benefit, particularly for patients at an advanced stage who have a very poor prognosis. Immunotherapy has changed the course of medical oncology, leading to potent antitumor efficacy in many types of solid cancers (8). Among the different developed immune therapeutic approaches, the use of immunomodulatory monoclonal antibodies (mAbs) targeting immune checkpoints has shown promising and durable clinical responses in several cancers, including some CRC (6). Encouraged by the recent success of immune checkpoint inhibitors (ICI), other immunotherapies for CRC patients are still in development, particularly approaches based on antitumor vaccination (9).

This review, focusing exclusively on human studies, highlights the close relationship between molecular features and the immune microenvironment in CRC. We discuss how this interaction helps to determine which patients are most likely to respond to immunotherapy and why some of them fail to respond. To illustrate this, we focus specifically on ICI, vaccines and adoptive T cell therapy (ACT). Here we face something of a paradox in that immune cell infiltration of primary non-metastatic tumor carries strong prognostic power for survival following resection, yet immune-based therapies have thus-far proven disappointing for the majority of CRC patients. A better understanding of the plethora of interactions between immune cells and the different genetic types of CRC should clarify some aspects of this discrepancy, as reviewed below.

## Immune Microenvironment in CRC

### Immune Players Associated With Good Prognosis

The composition of immune cells within CRC tumors that are infiltrated is heterogeneous and the key players are continuously subject to microenvironmental changes. T cells exhibit important antitumor activities and play an essential role in tumor control. Indeed, stronger expression of genes coding for components of Th1 (TBX21, IRF1, and Interferon-γ) and CD8 T cell pathways (CD8α, granzyme B, granulysin) has been documented in CRC tumors without signs of early metastasis relative to tumors with such signs (10). In addition, the expression of these genes was found to be inversely correlated with tumor recurrence (11). Following publication of these findings, numerous studies linked high proportions of infiltrating CD8 and CD4 T cells, and more particularly Th1 cells, with better prognosis in CRC patients (12–14). Consistent with these observations, a high degree of infiltration of dendritic cells (DCs) into tumors was reported mostly to be associated with more favorable clinical outcome (15, 16). However, well-known for their plasticity, DCs can acquire a tolerogenic phenotype when they mature in surroundings like the tumor microenvironment (TME), enriched in immunosuppressive cytokines (17). This may in part explain the association of DCs with poor prognosis reported in some CRC studies (18, 19). Infiltrating natural killer (NK) cells can also be negatively influenced by the immunosuppressive TME. This type of lytic effector cell is involved in the recognition and elimination of tumor cells by cytotoxic activity which is finely regulated by multiple activating and inhibitory receptors. As might be expected, several studies have shown that an extensive intratumoral infiltration of NK cells had a good prognostic impact in CRC (13, 20, 21). Nonetheless, an alteration of NK cell phenotype characterized by a decreased expression of activating receptors concomitant with an increased expression of inhibitory receptors has been shown to lead to the impairment of their cytotoxic functions, in both tumor and peripheral blood of CRC patients, thus also likely contributing to tumor escape from immunosurveillance (22–24).

In addition to the documented importance of the major CD4 or CD8 T cell types with an αß T cell receptor (TCR), there is a subset of mostly CD4/CD8 double-negative T cells carrying an alternative γδ TCR. These cells can be divided into two major subsets based on their δ chain type: Vδ1 T cells have a regulatory phenotype whereas Vδ2 T cells display inflammatory properties (25). While the role of γδ T cells within tumor-infiltrating lymphocytes (TILs) remains rather unclear, some recent studies have yielded new insights regarding their impact in CRC. A higher level of Vδ1 T cells and a lower percentage of Vδ2 T cells were reported in the tumor tissues as compared to the adjacent healthy tissues of patients with rectal cancer. This discrepancy in term of distribution, together with a positive and negative correlation of Vδ1 and Vδ2 T cells, respectively, with disease tumor stage, suggests a differential role of γδ T cell subsets (26). Similar results were reported by Meraviglia et al. in CRC patients, showing that both tumor-infiltrating γδ T cell subsets had an effector phenotype with reduced capacity to produce IFN-γ compared to that in the adjacent normal tissue (27). Moreover, γδ TILs and TCRGV9 gene expression revealed that γδ T cells and specific Vγ9Vδ2 T cells (the main subset of Vδ2 T cells) were correlated with disease-free (27) and overall survival (DFS and OS) in CRC (28). However, as γδ T cells were both Vδ1 and Vδ2 T cells, the impact of Vδ1 T cells on clinical outcome is still unclear in CRC so far. This remains an area of intense investigation.

### Immune Players Associated With Poor Prognosis

Multiple immunosuppressive immune cells are also commonly present in TME in CRC. Myeloid-derived suppressor cells (MDSCs) are a heterogeneous population of immature myeloid cells which can be identified by phenotypes, such as CD14^+^ CD11b^+^ CD33^+^ HLA-DR^*low/*–^. These cells are commonly found in many different tumors (29). CRC tumor cells promote the induction of immunosuppressive MDSCs which further facilitate CRC tumor development by releasing factors such as TGF-β, arginase, nitric oxide or reactive oxygen species (30). Indeed, the frequency of MDSCs is higher in both blood and tumor of CRC patients relative to the blood of healthy volunteers (30, 31), and an increased level is correlated with advanced tumor stage and metastasis in CRC (32). Moreover, patients with a high proportion of MDSCs were found to have a significantly shorter progression-free survival (PFS) on chemotherapy (14). Several studies have confirmed that MDSCs derived from the blood of CRC patients are able to inhibit the proliferation of autologous T cells *in vitro* (30, 32) and that blocking MDSC function restored the secretion of IFN-γ by T cells (33).

In addition to MDSCs, tumor-associated-macrophages (TAMs) play a central role in the modulation of immune function in the TME. TAMs are divided into two major distinct subsets based on their phenotype and function. M1 macrophages are involved in the control of tumor growth by secreting high levels of pro-inflammatory cytokines such as TNF-α, IL-1-β or IL-12 and by driving a potent Th1 response. Conversely, M2 macrophages are characterized by the production of arginase 1 and immunosuppressive cytokines such as IL-10 and TGF-β, which promote tumor progression, metastasis and angiogenesis (34). Both M1 and M2 macrophages are identified as CD14^*low*^ CD16^*high*^ CD68^+^ cells but can be distinguished by their differential expression of specific markers such as nitric oxide synthase 2 (NOS2), CD86, HLA-class II, and CD163, CD206, for M1 and M2, respectively (35). Contrary to other cancer types, the prognostic impact of TAMs in CRC remains controversial. Some reports associated a high proportion of TAMs with good prognosis but these studies characterized TAMs only using CD68 which does not allow M1 or M2 discrimination (36, 37). The assessment of the clinical impact of each subset revealed that, consistent with expectations, M1 macrophages are linked with a favorable clinical outcome (38) while increased densities of M2 macrophages are associated with a poor prognosis (39, 40). However, also here, some studies yielded conflicting results with the exact opposite effect of both M1 and M2 macrophages on clinical outcome (41, 42). This discrepancy could be explained in part by the high plasticity between macrophage subsets and by a lack of standardized markers to detect them, being different in different studies (43).

Similar to TAMs, the role of regulatory T cells (Tregs) in CRC has not been fully elucidated. Tregs are involved inter alia in the suppression of inflammation mediated by effector T cells by several mechanisms including the release of TGF-β and IL-10 (44). In CRC, the average amount of Tregs was found to be increased in the blood of patients relative to healthy volunteers, and in the tumor relative to the adjacent non-tumor tissue (45, 46). Moreover, several studies demonstrated that Tregs derived from both blood and tumor of CRC patients were able to suppress the proliferation of autologous CD4 and CD8 T cells (47, 48), and that the frequency of Tregs was negatively correlated with the expression of IFN-γ and IL-2 in the tumors (49). Despite these observations, the impact of Tregs on prognosis in CRC is still unclear, as some studies have linked them to a poor prognosis (40, 50) while others have reported that their presence predicts a favorable outcome (51, 52). A likely explanation of these conflicting reports could be the co-existence of phenotypically similar Treg subsets which nonetheless have different functions. Lin et al. identified two subsets of Tregs based on Foxp3 and CD45RA expression which were increased in CRC patients: strongly suppressive activated Tregs (Foxp3^*high*^CD45RA^–^) and non-suppressive Tregs (Foxp3^*low*^ CD45RA^–^). While activated Tregs were found to inhibit CD4 T cell proliferation and to highly express CTLA-4, non-suppressive Tregs did not prevent the proliferation of CD4 T cells and were characterized by secretion of a large amount of inflammatory cytokines including IFN-γ, IL-2 and TNF-α (53). That is, the latter were not actually functional Tregs. Later, Saito et al. corroborated these findings and demonstrated that only activated Tregs were associated with advanced stage CRC and a poor prognosis (54). These studies clearly show that using Foxp3 as the sole marker to characterize Tregs is not sufficient and its use might be the reason for contradictory findings on the role of Tregs in CRC.

Finally, Th17 cells that are endowed with strong inflammatory properties by virtue of their IL-17 and IL-21 production, were found at higher proportions in the tumor as well as in the blood of CRC patients relative to healthy volunteers (45, 49). In addition, a high amount of Th17 was associated with tumor progression and a poor prognosis (12, 13, 55). While the mechanisms involving Th17 in CRC are not completely understood, these cells could act through the release of IL-17 as poor prognosis has been reported for patients with high levels of IL-17 (56, 57).

### Immunoscore: A Strong Predictor of Clinical Outcome

All these immune cell subsets form a complex network and cross-talk in different ways within the tumor. Their location rather than mere presence is likely to be of crucial importance. They can be located in the core of the tumor and therefore directly interact with malignant cells or in the periphery of the tumor, in the invasive margin, or excluded altogether. The analysis of the location, density, nature and functional orientation of immune cells defines the immune “contexture” within the tumor (58). The immune contexture is heterogeneous according to tumor types, varies between patients with the same malignancy and affects patients’ survival, as previously mentioned. Based on this observation, a score predicting the clinical outcome of patients has been developed. The “Immunoscore” is based on the quantification of two lymphocyte populations defined as CD3^+^/CD45RO^+^ or CD8^+^/CD45RO^+^ or CD3^+^/CD8^+^, in both tumor core and invasive margin. A score of 0 is characterized by a low density of both types of cell in both tumor regions whereas a score of 4 identifies tumors with a high density of these cells. Between 0 and 4, a high density of one marker in one of the two tumor regions gives a score of 1 for example (59). Patients with stage I-II CRC who underwent primary resection experienced different outcomes in term of DFS and OS, according to their Immunoscore. Patients with a score of 4 (high densities of CD45RO^+^ and CD8^+^ cells) were at low risk, with 5-year OS of 86.2%. In contrast, patients with a score of 0 (low densities of CD45RO^+^ and CD8^+^ cells) were at higher risk with a 5-year OS of only 27.5% (7). In the same way, the prognostic value of the Immunoscore on the clinical outcome of patients was confirmed in patients with advanced CRC stages (60). Strikingly, it has been shown that even stage I patients with low infiltrations of CD3^+^ cells and CD45RO^+^ cells in both tumor core and invasive margin have a poor prognosis in term of DFS, similar to that of patients with the worst stage IV disease prognosis according to the UICC-TNM classification (11). These observations led the authors to investigate the prognostic power of the immune score as compared to the UICC-TNM classification. Multivariate survival analyses combining UICC-TNM stages with the Immunoscore revealed that only the Immunoscore remained significantly associated with DFS and OS, whereas the UICC-TNM classification became non-significant (60). These data clearly showed that the Immunoscore has a higher prognostic value than the UICC-TNM classification.

Since the validation of the Immunoscore as a new component of a TNM-Immune classification of cancer (61), several teams have worked on its improvement by suggesting the inclusion of other markers. PD-L1 expression was found to be significantly associated with a high Immunoscore (62, 63) and a combined survival analysis revealed 4 distinct groups of patients with significant differences in the OS, refining survival groups defined only using the Immunoscore (64). Further refinement is certainly to be expected.

## CRC Classification: Association of Molecular and Immune Profiles

### Molecular Phenotypes of CRC

It is now clear that CRC is not a single unique disease but presents several heterogeneous and complex subtypes, each characterized by different genetic and epigenetic alterations. The three major pathways of genomic instability leading to CRC development involve chromosomal instability (CIN), the CpG-island methylator phenotype (CIMP) and MSI (65).

CIN tumors are observed in 85% of CRC and harbor mutations in the tumor suppressor genes APC, TP53, SMAD2/4 and DCC, and the proto-oncogenes KRAS, CTNNB1 and PIK3CA (66). Hyperactivation of the WNT signaling pathway is observed in these tumors, usually arising from mutations in the APC gene. CIN tumors are characterized by losses or gains of portions of chromosomes or entire chromosomes, resulting in an abnormal number of chromosomes and a loss of heterozygosity. These events are caused by mutations in genes involved in chromosome segregation such as BUB1 or BUBR1, the formation of centrosomes such as AURKA and PLK1, telomere formation and DNA damage response such as TP53 and BRCA1/2 (66). Another pathway of CRC tumorigenesis involves hypermethylation of CpG island sites which are commonly found in the promoters of many genes, resulting in gene silencing. CIMP tumors in particular display aberrant promoter methylation of tumor suppressor genes or other tumor-related genes (67). It has been shown that CIMP is associated with the hypermethylation of the MLH1 gene promoter leading to MSI, the third major phenomenon related to genomic instability (68).

Approximately 15% of CRC have MSI caused by a defect in MMR activity. The MMR system is composed of several heterodimers including MLH1/PMS2 and MSH2/MSH6 that recognize and correct wrong insertions, deletions or mis-incorporated bases during DNA replication, which otherwise would result in mismatches between the two DNA strands (5). DNA-polymerase that is responsible for reading DNA during replication is more likely to slip and make errors in regions with short tandem repeat sequences such as microsatellites. Deficiencies in MMR result in increased accumulation of genetic errors in these repeated sequences and consequently lead to the development of MSI tumors that are hypermutated (65). Most MSI tumors are sporadic due to epigenetic silencing of MMR genes such as the hypermethylation of the MLH1 promoter related to CIMP. MSI can also occur in patients with Lynch Syndrome due to germline mutations in one of the MMR genes MLH1, PMS2, MSH2, or MSH6 (5, 69). In contrast to MSI tumors that display deficient MMR (dMMR), tumors with proficient MMR (pMMR) are MSS tumors and have markedly different characteristics, especially regarding neoantigen expression.

### Consensus Molecular Subtypes Display Distinct Immune Profiles

**FIGURE 1:**
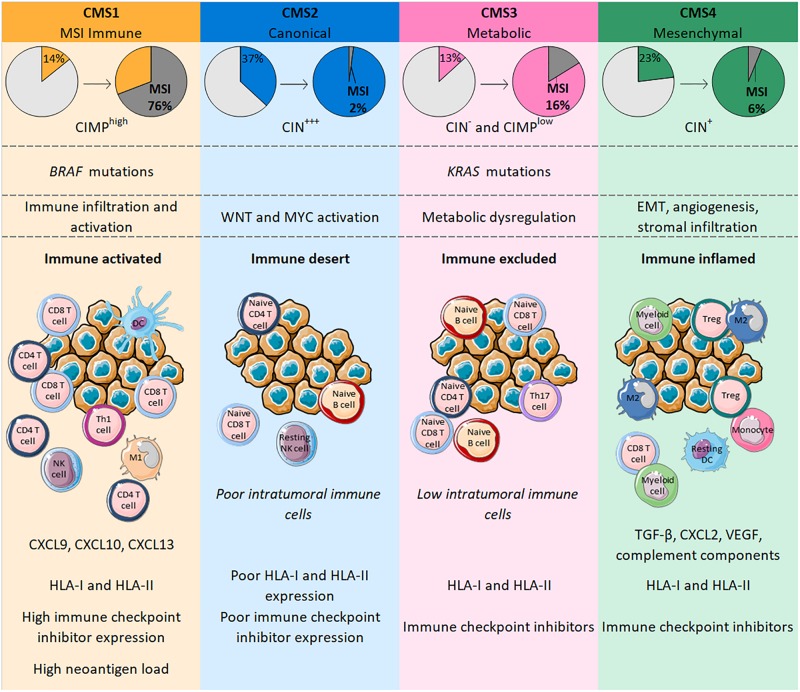
CMS in CRC are classified according to genetic modifications and intratumoral immune phenotype, with distinct profiles. CMS1 is highly enriched in MSI tumors bearing BRAF mutations whereas CMS4 tumors only number a few MSI cases and are characterized by an EMT associated with strong stromal activity and angiogenesis. Both CMS1 and CMS4 tumors are heavily infiltrated with immune cells that confer a specific functional immune landscape. While CMS1 tumors are enriched with activated CD4 and CD8 T cells, express high levels of HLA and immune checkpoints and have a high neoantigen load; CMS4 tumors display an unfavorable inflamed immune environment characterized by TGF-β, VEGF, complement components and an infiltration mainly driven by immunosuppressive cells (Tregs, M2 macrophages, myeloid cells). CMS2 and CMS3 gather tumors with upregulated WNT and MYC signaling pathways and tumors with profound metabolic dysregulation together with KRAS mutations, respectively. Their immune phenotype is similar with a poor/low infiltration of immune cells that are mostly naïve cells. Contrary to CMS2 tumors, CMS3 tumors maintain HLA and ICI expression.

The different genetic and epigenetic events found in CRC led 6 independent teams to propose various CRC molecular subtyping systems. There are few similarities between these classifications, and the number of subtypes reported varied from 3 to 6 that did not allow a single classification system (70–75). Subsequently, the CRC Subtyping Consortium was formed to evaluate the results of CRC subtyping algorithms, previously defined by the 6 teams. Following the normalization of these data, they described 4 consensus molecular subtypes (CMS), each with distinct molecular and immune features (76) (Figure 1).

Consensus molecular subtypes 1 (14% of all CRC) includes tumors frequently characterized by possessing BRAF mutations, highly enriched in CIMP and MSI tumors (76%) reflecting a hypermutated phenotype (76). Indeed, MSI-High tumors have a mutational rate 20 times higher than MSS tumors and more than 80% of MSI-High tumors display a high tumor mutation burden (TMB) of >20 mutations/Mb (77). The TMB is positively correlated with the number of neoantigens in a large range of cancers, including CRC (78). Similar to the mutational rate, a recent study showed that the median number of neoantigens in CRC patients with MSI-High tumors was around 20 times higher than in MSS tumors (79). Neoantigens are newly formed antigens resulting from tumor somatic mutation that confer tumor immunogenicity and can elicit an antitumor immune response (80). Accordingly, CMS1 tumors are highly infiltrated with immune cells and particularly with activated lymphocytes. Indeed, CMS1 shows high levels of CD8 T cells, memory CD4 T cells, Th1 and T follicular helper cells, γδ T cells, and also activated DCs, NK cells and M1 macrophages (76, 81, 82). This immune landscape confers an inflamed environment to CMS1 tumors which is in line with decreased amounts of Tregs, monocytes and resting NK and CD4 T cells relative to other CMS categories (76, 81). Interestingly, CMS1 tumors strongly express genes involved in T cell chemotaxis such as CXCL9 and CXCL10; genes specific for homeostasis and activation of both T and NK cells, respectively, IL-15 and IFN-γ; and the gene coding for CXCL13, a B cell-attracting chemokine, that is also implicated in the formation of tertiary lymphoid structures (TLS) (82). TLS share many similarities with lymph node structures where immune responses normally occur; high TLS densities were observed in CRC, particularly within MSI tumors (83, 84). Along with the upregulation of genes belonging to HLA class I and class II families and those involved in antigen processing and presentation (TAP1, TAP2, β2-microglobulin), all of these elements suggests strong antitumor immunity in the microenvironment of CMS1 tumors (81, 82). However, these tumors may escape from immune surveillance by expressing immune checkpoint molecules, with genes encoding PD-1, PD-L1, CTLA-4 or LAG3 transcribed in CMS1 tumors (76, 81, 82).

The “canonical” subtype CMS2 (37% of all CRC) includes CIN tumors displaying epithelial differentiation markers, together with upregulated WNT and MYC signaling pathways. Among the 4 CMS categories, CMS2 is the group with the fewest MSI tumors (only 1– 2%) (76). Unlike CMS1, CMS2 tumors have a poor intratumoral immune response characterized by low levels of lymphocytes, monocytes and myeloid cells. In line with this, these tumors possess few transcripts of genes coding for the chemokines implicated in T cell chemotaxis and activation, or of genes involved in antigen processing and presentation. Additionally, CMS2 tumors show a particularly poor expression of PD-1 and PD-L1 (76, 81, 82). The CMS2 subtype is often defined as being an “immune desert” and the few immune cells found within these tumors are resting NK cells, naive CD4 T cells or B cells that are not able to mediate active antitumor immunity in this context (81).

The “metabolic” subtype CMS3 (13% of all CRC) comprises tumors with frequent KRAS mutations and some cases of MSI (16% of CMS3). Gene expression analysis of CMS3 showed a profound metabolic dysregulation in many pathways (76). The immune landscape of CMS3 tumors was designated “immune excluded” and is similar to CMS2 with poor infiltration of lymphocytes, monocytes and myeloid cells. Nonetheless, these tumors are enriched in cells expressing PD-1, Th17 cells, naive B and T cells, and resting T cells, indicating a dormant immune microenvironment. The expression of HLA class I and class II seems to be maintained but probably differs according to the heterogeneity of tumors (76, 81, 82).

Finally, the CMS4 “mesenchymal” group (23% of all CRC) encompasses tumors characterized by an epithelial-mesenchymal transition (EMT) associated with matrix remodeling, strong stromal activity, activation of the TGF-β signaling pathway and angiogenesis (76). MSI tumors account for only 6% of the CMS4 subtype. CMS4 tumors nonetheless have an inflammatory profile characterized by an enrichment of complement components and high levels of infiltrating lymphocytes and macrophages. However, these tumors display fewer CD8 and CD4 T cells and more Tregs than CMS1 tumors. Also, macrophages found in CMS4 tumors have a predominantly M2 phenotype, whereas the level of M1 macrophages is decreased, generating a protumoral microenvironment. These tumors also show a strong infiltration of monocytes, eosinophils, myeloid cells and resting DCs, whereas levels of activated DCs and NK cells are low (81, 82). This inflammatory environment supports the development of tumors through immunosuppressive and angiogenic factors such as TGF-β, CXCL12, or VEGF widely found in CMS4 tumors. Such factors can be produced by Tregs, fibroblasts or endothelial cells that constitute a large part of the cancer in CMS4 tumors. Despite the presence of immunosuppressive elements, the expression of both HLA and immune checkpoints is retained (81, 82).

## Immune Checkpoint Blockade in CRC

### Response to Immune Checkpoint Inhibitors: The Paradox of CRC

In the last few years, immunotherapy based on the reactivation of the host immune system has achieved unprecedented success as cancer therapy for many solid tumors. Indeed, activated CD4 and CD8 T cells express immune checkpoint receptors such as PD-1 or CTLA-4 that are frequently activated in the TME and responsible for inhibiting the immune response mediated by T cells. The use of checkpoint inhibitors to block immune checkpoint receptors and their ligands has yielded notable survival benefits in solid cancers widely infiltrated by immune cells, including melanoma and lung cancers (85–87).

Although the immune landscape differs according to CMS tumors, as discussed above, infiltrating immune cells have been identified as strong prognostic markers in CRC, suggesting a crucial role in tumor control and supporting the use of checkpoint inhibitors as therapeutic agents. However, initial approaches using anti-PD-1 mAbs in CRC were disappointing as only little, if any, clinical benefit was obtained. For example, in a phase I study assessing the efficacy of anti-PD-1 mAbs in patients with advanced solid tumors, a complete response was reported in only 1 of 14 patients with CRC (88). The tumor of this one responding patient displayed expression of PD-L1 by macrophages, lymphocytes and rare tumor cells and infiltrating CD3^+^ and PD-1^+^ T cells (89). Given that this tumor had an MSI phenotype, the authors hypothesized that this subtype might be predictive of response to ICI and a phase II study was conducted to compare the response to PD-1 inhibitor (pembrolizumab) in CRC patients with either MSI and MSS tumors (90). This clinical trial enrolled 32 patients comprising 11 and 21 CRC patients with dMMR and pMMR, respectively. The 20-week objective response and PFS rates were, respectively, 40% and 78% for CRC patients with dMMR tumors versus 0% and 11% for those with pMMR tumors. Consistent with this, a high number of potential mutation-associated neoantigens was identified in CRC patients with dMMRs tumor (mean of 578 versus 21) and was associated with prolonged PFS (90). These promising results led the US Food Drug Administration (FDA) to approve the use of pembrolizumab in patients with MSI metastatic tumors (of any histology). A different anti-PD-1 mAb, nivolumab, has also been studied in combination with ipilimumab (anti-CTLA-4 mAb) in an ongoing phase II study for CRC patients with dMMR. In that study, 12-month PFS and OS rates were 71% and 85% versus 50% and 73%, respectively, for nivolumab alone, suggesting a superior efficiency of the combination therapy (91). Interestingly, some CRC patients (2–3%) harbor MSS tumors with DNA polymerase epsilon or delta (POLE, POLD) mutations. It has been reported that in a subset of MSS tumors, mutations in the genes encoding these enzymes responsible for DNA synthesis and repair (92, 93) are associated with an ultramutated phenotype defined by a high number of frameshift mutations. Indeed, predicted neoantigen load is 3– 4 times higher in MSS tumors carrying POLE mutations than in MSI CRCs (13, 79). Interestingly, POLE-mutated CRCs show strong immune cell infiltration similar to MSI tumors, with particularly high levels of CD3 T, CD8 T and NK cells (94, 95). Jun et al. reported the first case in CRC of clinical response to pembrolizumab in one patient harboring a tumor with an MSS phenotype and POLE mutation. Multispectral fluorescent immunohistochemistry performed on the tumor revealed a large proportion of PD-1^+^ CD8^+^ T cells and marked infiltration of CD68^+^ TAMs expressing PD-L1 (96, 97). Such results were also observed in two patients with endometrial cancer and need to be further investigated, but they do suggest that not only MSI but also POLE mutations might be predictive markers in immune checkpoint blockade (98–100). Currently, three clinical trials are ongoing to investigate the clinical benefit of anti-PD-L1 mAbs in POLE-mutated CRC (NCT03435107, NCT03150706, and NCT03827044).

**FIGURE 2:**
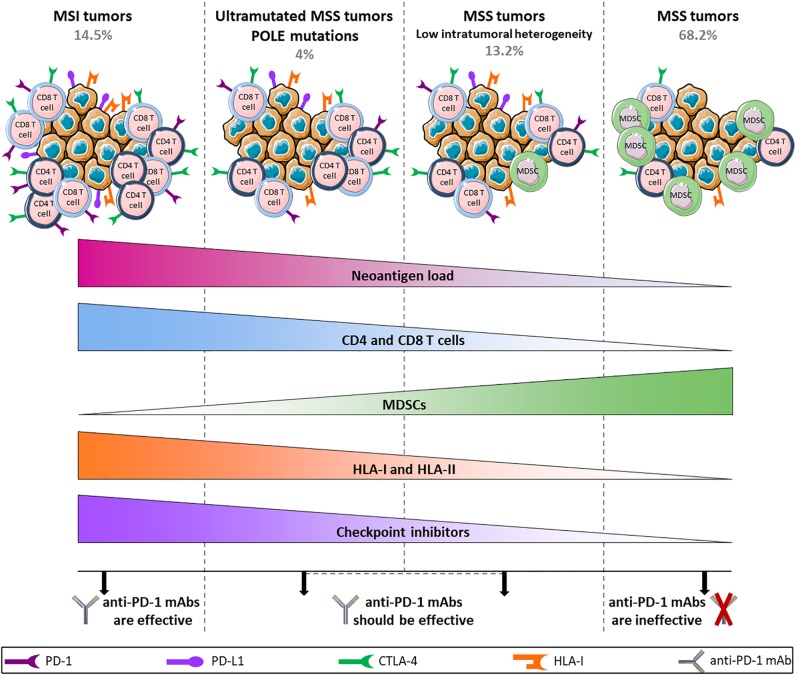
Hypothetical model of anti-PD-1 mAbs efficacy/inefficacy according to different subtypes of CRC tumors. MSI and ultramutated MSS tumors show high neoantigen load associated with a large infiltration of CD4 and CD8 T cells and an upregulation of immune inhibitory receptors, allowing the use of anti-PD-1 mAbs. In contrast, tumor escape mechanisms in MSS tumors rely on a downregulation of HLA class-I and class-II along with a high infiltration of MDSCs, and the level of T cells remains low. In addition, the weak expression of inhibitory receptors in these tumors does not allow the use of anti-PD-1 mAbs, apart from a restricted group of MSS tumors characterized by low intratumoral heterogeneity that shares several features with mutated tumors and that should be able to respond to anti-PD-1 mAbs.

Despite this tremendous breakthrough regarding the use of checkpoint inhibitors in the treatment of MSI metastatic tumors, 85% of CRC cases are MSS tumors with low TMB for which immune checkpoint blockade fails to elicit a response. This discrepancy is not consistent with expectations from the Immunoscore which represents a strong predictor of clinical outcome at primary resection in CRC, irrespective of the microsatellite status (61, 101). Angelova et al. provided some explanations for this paradox by analyzing the immunophenotypes and antigenomes of 475 CRC tumors. Based on intratumoral heterogeneity, 6 groups were identified with their own features including MSI and hypermutated MSS groups (POLE mutations) that displayed greater intratumoral heterogeneity and 4 other MSS groups with low, two intermediate and high heterogeneity. As expected, MSI and hypermutated MSS groups showed high TMB and neoantigen loads, an enrichment of activated CD4 and CD8 T cells and conversely a depletion of MDSCs and Tregs and an increased expression of immune checkpoint receptors such as CTLA-4 and PD-1 and its ligand PD-L1. Surprisingly, differences were observed within MSS groups. MSS tumors were characterized as enriched for MDSCs, poorly infiltrated by T cells and downregulated checkpoint inhibitors and HLA class-I and class-II. The latter showed similar features to hypermutated groups, although the amount of CD4 and CD8 T cells was lower (13). These data suggest different tumor escape mechanisms depending on the type and heterogeneity of tumors that are directly related to the effectiveness of anti-PD-1 mAbs (Figure 2). Indeed, by binding to PD-1 expressed by T cells, anti-PD-1 mAbs prevent the interaction between PD-1 and PD-L1 that is upregulated in MSI and hypermutated MSS tumors. However, even if MSS tumors are infiltrated by T cells, they will not respond to therapy with anti-PD-1 mAbs as they do not express ICI but escape from immunosurveillance, for example, by downregulating HLA expression. Importantly, this study highlights that a particular fraction of non-hypermutated MSS tumors might in theory respond to PD-1/PD-L1 blockade therapy. This is in accordance with Giannakis et al. who speculate that a subset of MSS POLE-wild-type tumors could be responsive to checkpoint inhibition based on a significant association between high neoantigen load and high number of TILs found in this subset (79).

These observations make the development of new strategies for accurately predicting responses to ICI of paramount importance. In the last few years, much effort has been expended on the development of patient-derived organoids (PDOs) as novel pre-clinical cancer models for examining drug responses, for example (102). Tumor organoids are 3-dimensional structures derived from primary tumor cells and cultured *in vitro* on an extracellular matrix substitute in serum-free medium containing growth factors. Interestingly, PDOs can closely recapitulate the structure and function of the original tumor. In addition, the genetic heterogeneity in the composition of the primary tumor is generally conserved within PDOs (103, 104). Thus, in CRC, it has been found that PDOs and the uncultured primary tumor shared 90% of somatic mutations including mutations in driver genes (105). This preservation of genetic diversity along with their other features suggests that PDOs are likely to represent a better model of individual tumors than standard cancer cell lines. Importantly for routine application, it has been shown that the establishment of organoids derived from CRC primary tumor currently has a success rate as high as 71–90%, in contrast to only around 10% for the generation of CRC cell lines (102). As hypothesized, several independent studies have demonstrated that PDOs accurately predicted responses to chemotherapy (106, 107) and anticancer agents (103, 104, 108). Remarkably, Vlachogiannis et al. reported 88% positive and 100% negative predictive responses to various agents and chemotherapy, between results found in PDOs and in patients with CRC or gastroesophageal cancer (109). However, while PDOs faithfully represent the original tumor cells, the lack of an intact TME including fibroblasts and immune cells, hampers their use for predicting responses to immunotherapy. To overcome such barriers, Neal et al. developed a novel approach combining generation of PDOs with air-liquid interface (ALI) culture systems which allow the preservation and propagation of immune cells and fibroblasts from primary tumors within their matching organoids, for several weeks. The structure and mutation spectrum were well-preserved in ALI PDOs derived from a wide range of cancer subtypes (including CRC, RCC, lung, or pancreatic cancers); numerous immune cell subsets were retained (CD4 T cells, CD8 T cells, B cells, NK cells, M2 macrophages, Tregs, and exhausted T cells); and T cell clonal diversity was also maintained. Treatment of ALI PDOs with nivolumab led to T cell activation, expansion and cytotoxicity and a loss of PD-1 expression by some of the tumor cells. Remarkably, nivolumab response rates in ALI PDOs were similar to those found in clinical trials, in different cancer types (110). These findings suggest that ALI PDOs could be a powerful tool for predicting responses to immune checkpoint blockade. Further investigations will be needed to determine whether ALI PDOs could have a predictive value for response to immunotherapy in CRC, which was a tumor type not included in the study by Neal et al. This model will probably allow to in-depth investigation to understand the differences in responses to ICI between MSI and MSS CRC.

### Mechanisms of Resistance to Immune Checkpoint Inhibitors Associated With Immune Escape in MSI Tumors

The data reviewed above strongly support the notion that all MSI tumors should respond to ICI, but sadly this is not the case in practice due to multiple different tumor escape mechanisms, and a significant fraction of patients has progressive disease. Indeed, some patients are initially resistant or develop resistance and fail to respond to immune checkpoint blockade (111). The mechanisms responsible for resistance are not fully understood but functional alterations have been identified in some pathways. One well-known mechanism by which tumor cells escape from immune surveillance is impairment of the antigen processing machinery (APM) or in the expression of the HLA complex [HLA class I heavy chain or β2-microglobulin (B2M)], leading to defective antigen processing and presentation. Early studies investigated mutations of the B2M gene in CRC and showed that B2M mutations were significantly associated with the MSI phenotype and less prevalent in MSS tumors (112, 113). Consistent with this, resistance to anti-PD-1 mAb was described in CRC patients with MSI tumors carrying B2M mutations (114). Accordingly, Janikovits et al. found significantly higher infiltration of PD-1^+^ T cells within B2M-mutants than into B2M-wild type MSI tumors, suggesting that the loss of HLA class I expression mediated by B2M mutation is an immune evasion mechanism that happens in an environment enriched in activated PD-1^+^ T cells (115). In addition, Giannakis et al. highlighted HLA mutations in MSI CRCs that mostly occurred in the peptide binding domain, suggesting ineffective presentation of antigens by the HLA complex. They also found that 40 and 85% of tumors with high numbers of TILs also had mutations in HLA genes and in genes involved in APM including TAP1 and TAP2, respectively (79). Such results have been confirmed and extended by two recent studies. In addition to B2M mutations and TAP1/TAP2 mutations in MSI tumors, mutations in HLA-A, HLA-B and HLA-C genes that were mostly identified as truncating mutations were found (116, 117). Mutations affecting the gene encoding the HLA class I transactivator NLRC5 were found to be associated with decreased expression of HLA class I (116, 117). In addition, Grasso et al. identified a mutation in the RFX5 gene involved in transcriptional regulation of HLA class I genes, together with NLRC5. As expected, tumors carrying RFX5 mutations displayed low levels of HLA class I expression, suggesting a functional significance for both NLRC5 and RFX5 for HLA class I expression in MSI tumors (117). Mutations in RFX5 and to a lesser extent CIITA were also found in HLA class II-negative MSI tumors (118, 119). Moreover, MSI tumors harboring RFX5 mutations were characterized by high infiltration of T cells (115, 119). Taken together, these data suggest that MSI CRCs are subject to strong immune selection pressure, leading to the development of escape variants involving alterations of antigen processing and presentation pathways.

Another immune evasion mechanism commonly relies on mutations in JAK1 and JAK2 genes. JAK1 and JAK2 are kinases downstream of the IFN-γ receptor and are both required to mediate IFN-γ signaling. Disruption of genes coding for these kinases was found in melanoma and CRC patients with primary resistance to PD-1 blockade (120). Surprisingly, a CRC patient who failed to respond to PD-1 inhibition had an MSI phenotype along with high mutational load and no APM or HLA complex; however, a homozygous truncating JAK1 mutation resulting in loss of protein function was reported in this patient (120). In MSI CRC patients, JAK1 mutated tumors exhibited a lower IFN-γ gene expression signature than wild-type tumors. Additionally, PD-L1 gene expression was significantly down-regulated in tumors carrying JAK1 mutations (121). Such results were also found in other cancer subtypes, including melanoma, endometrial and stomach cancers (120, 122, 123). Garcia-Diaz et al. showed that PD-L1 expression on melanoma cell is regulated by the IFN-γ receptor signaling pathway through JAK1 and JAK2 and several STATs that lead to the binding of IRF1 to the PD-L1 promoter (124). Thus, tumor cells can escape IFN-γ-mediated immune responses through JAK1/JAK2 mutations leading to the loss of IFN-γ signaling and consequently preventing PD-L1 expression.

Interestingly, it has also been shown that MSI tumors display heterogeneity in relation to their immune microenvironment. A study compared sporadic dMMR and Lynch-associated dMMR tumors in CRC patients and highlighted a significantly higher number of CD3 and CD8 TILs in both the invasive margin and the center of tumor in Lynch-associated dMMR patients. Additionally, these patients possessed more somatic mutations and neoantigens than patients with tumors harboring sporadic dMMR (*P* = 0.006 and *P* = 0.009, respectively) (125). Sveen et al. reported that MSI tumors from CMS1 were characterized by a significantly higher level of infiltrating immune cells including cytotoxic T lymphocytes, DCs and monocytes, and strong PD-1 signaling, compared with MSI tumors from CMS2-4. They also showed a higher TMB in MSI tumors from CMS1 than those from the other CMS subtypes (*P* = 0.03), supporting the notion that MSI tumors from CMS1 are the most immunogenic (121). These observations could explain, at least in part, disease progression in a significant subset of patients with MSI tumors having received checkpoint inhibitors. Although the clinical outcome of patients enrolled in clinical studies blocking immune checkpoints does not seem to be influenced by Lynch Syndrome so far, no study seems to have investigated the association between CMS and response to checkpoint inhibitors yet (55, 126).

### Future Alternatives: New Generation of Immune Checkpoint Inhibitors

Success of immune checkpoint therapy and particularly PD-1/PD-L1 pathway blockade highlights the critical role of immune inhibitory receptors in tumor evolution. However, tumor resistance to anti-PD-1 mAbs and tumors for which theses mAbs are ineffective remain high, particularly in CRC. Therefore, alternatives need to be investigated to allow more patients to benefit from immunotherapy. Recently, interest in other immune checkpoints has intensified and novel potential targets have been identified such as TIM-3, LAG-3, TIGIT, or VISTA. Expressed on activated T cells and NK cells among others, these receptors have been found highly expressed on TILs as compared to circulating T cells in CRC patients (127–130). As with PD-1 or CTLA-4, physiologically, these inhibitory receptors are induced following the activation of T cells to prevent overstimulation, which may result in an exhausted state. PD-1 was particularly found to be commonly co-expressed with TIM-3 (131, 132), LAG-3 (133, 134), TIGIT (135), and VISTA (136) on infiltrating T cells in several cancer types including renal cell carcinoma (RCC), melanoma, ovarian, gastrointestinal and non-small cell lung cancers (NSCLC). In CRC, Xu et al. showed that the level of tumor-infiltrating CD8 T cells producing IFN-γ was greatly reduced when they express both PD-1 and TIM3 as compared to PD-1^–^ TIM-3^–^ or PD-1^+^ TIM-3^–^ CD8 T cells, suggesting a more dysfunctional state of PD-1^+^ TIM-3^+^ CD8 T cells than PD-1^+^ TIM-3^–^ CD8 T cells (137). Similarly, the amounts of PD-1^+^ TIM-3^+^ CD8 T cells expressing CD107, granzyme B and IFN-γ (138) and PD-1^+^ LAG-3^+^ CD8 T cells producing IFN-γ and TNF-α (133) were decreased as compared to their counterparts without inhibitory receptors, in ovarian cancer. In line with this, Laheurte et al. found a lower levels of telomerase (TERT)-specific PD-1^+^ TIM-3^+^ T cells than TERT-specific PD-1^+^ TIM-3^–^ or TIM-3^+^ PD-1^–^ T cells in the blood of NSCLC patients, indicating a much more impaired functionality of PD-1^+^ TIM-3^+^ T cells (139). Based on these observations, it might be expected that the use of single agent anti-PD-1 mAbs is not always sufficiently effective to restore functionality of T cells, particularly when these latter co-express various other immune inhibitory receptors. The rationale of targeting PD-1 with the new generation of inhibitory receptors has led to the implementation of clinical studies. These studies are still ongoing and investigate TIM-3, LAG-3, TIGIT, and VISTA as potential therapeutic targets, either alone or in combination with anti-PD-1 mAbs in advanced cancer including CRC (Table 1). Preliminary data showed that 11 of 27 patients treated with anti-LAG-3 antibody (REGN3767) had stable disease whereas combination therapy with an anti-PD-1 (REGN2810) led to partial responses in 2 of 42 patients. Partial responses were also observed in 2 of 12 patients previously included in the group of monotherapy and who subsequently received REGN2810 (140). Moreover, data from other clinical trials indicated that both anti-TIM-3 antibodies LY3321367 and MBG453 were well tolerated as a monotherapy and in combination with anti-PD-L1 (LY3321367) or anti-PD-1 (PDR001) antibodies, respectively (141, 142). In NCT02608268, 25 of 87 and 34 of 86 patients treated with MBG453 alone and in combination with PDR001, respectively, showed stable disease. Encouragingly, 4 partial responses were observed in patients having received the combination therapy, including 2 CRC patients (6 CRC among 86 patients) (141). So far, the expression of TIM-3, LAG-3, TIGIT, and VISTA has not been extensively studied with respect to microsatellite status. The use of the new generation of checkpoint inhibitors in clinical studies should reveal if they can be more effective in MSS than anti-PD-1 mAbs; results are eagerly anticipated.

**TABLE 1:** Overview of some of the ongoing clinical trials investigating TIM-3, LAG-3, TIGIT, and VISTA inhibitors in advanced cancers including CRC.

**ClinicalTrial.gov identifier**	**Tumor type**	**Phase**	**Intervention**	**Enrollment status**
**anti-TIM-3**				

NCT03652077	Multiple advanced cancers including CRC	I	**INCAGN02390**	Recruiting
NCT03489343	Advanced solid tumor malignancies or lymphoma	I	**Sym023**	Recruiting
NCT03311412	Advanced solid tumor malignancies or lymphoma	I	**Sym023** + Sym021 (anti-PD-1)	Recruiting
NCT03099109	Advanced relapsed/refractory solid tumors	I	**LY3321367** ± LY3300054 (anti-PD-Ll)	Recruiting
NCT03744468	Advanced solid tumors	l/ll	**BGB-A425** + tislelizumab (anti-PD-1)	Recruiting
NCT02608268	Advanced malignancies	l/ll	**MBG453** ± PDR001 (anti-PD-1)	Recruiting
NCT02817633	Advanced solid tumors melanoma, NSCLC and CRC	I	**TSR-022** + TSR-042 (anti-PD-1) + TSR-033 (anti-LAG3) or **TSR-022** + TSR-042	Recruiting

**anti-LAG3**				

NCT02060188	Recurrent or metastatic MSI-H and non-MSI-H CRC	II	**Relatlimab** + nivolumab (anti-PD-1)	Active, not recruiting
NCT03642067	MSS advanced CRC	II	**Relatlimab** + nivolumab	Recruiting
NCT03607890	MSI-H solid tumors	II	**Relatlimab** + nivolumab	Recruiting
NCT02966548	Advanced solid tumors	I	**Relatlimab** ± nivolumab	Recruiting
NCT03335540	Advanced solid tumors	I	**Relatlimab** + nivolumab	Recruiting
NCT03459222	Advanced solid tumors	l/M	**Relatlimab** + nivolumab + BMS-986205 (IDO1 inhibitor) or **Relatlimab** + nivolumab + ipilimumab (anti-CTLA-4)	Recruiting
NCT03538028	Multiple advanced cancers including MSI-H CRC	I	**INCAGN02385**	Recruiting
NCT03489369	Advanced solid tumor malignancies or lymphoma	I	**Sym022**	Recruiting
NCT03311412	Advanced solid tumor malignancies or lymphoma	I	**Sym022** + Sym021	Recruiting
NCT03250832	Advanced solid tumors	I	**TSR-033** ± anti-PD-1	Recruiting
NCT02817633	Advanced solid tumors	I	**TSR-033** + TSR-042 + TSR-022	Recruiting
NCT02720068	Advanced solid tumors	I	**MK-4280** ± pembrolizumab (anti-PD-1)	Recruiting
NCT03005782	Advanced malignancies	I	**REGN3767** ± REGN2810 (anti-PD-1)	Recruiting

**anti-TIGIT**				

NCT03628677	Multiple advanced cancers including CRC	I	**AB154** ± AB122 (anti-PD-1)	Recruiting
NCT03119428	Locally advanced or metastatic solid tumors	I	**OMP-313M32** ± nivolumab	Active, not recruiting
NCT02794571	Locally advanced or metastatic tumors	I	**MTIG7192A** ± atezolizumab (anti-PD-Ll)	Recruiting
NCT02913313	Advanced solid tumors	l/M	**BMS-986207** ± nivolumab	Recruiting

**anti-VISTA**				

NCT02671955	Advanced cancers	I	**JNJ-61610588**	Terminated

*Bold treatments correspond to anti-TIM-3, anti-LAG3, anti-TIGIT or anti-VISTA mAbs.*

## Tumor-Associated Antigens and Neoantigens in CRC Vaccination

Cancer vaccination is another immunotherapeutic strategy used in CRC and has traditionally targeted TAAs that are significantly over-expressed by cancer cells relative to normal cells. There is good reason to believe that eventual combination therapies including bother ICI and cancer vaccines may yield synergistic effects. The concept of antitumor vaccination is based on the establishment of TAA-specific antitumor immune responses that can eliminate tumor cells expressing these antigens. Several types of vaccine formulations have been investigated in CRC including autologous, DC, viral vector, and peptide-based vaccines. Briefly, autologous vaccines are produced with cells removed from a patient’s own tumor and therefore contain the whole patient-specific TAAs (143). DC vaccine development involves harvesting DCs from the patients, pulsing them *ex vivo* with TAAs or tumor cell components, for example, and re-infusing them into the patients after their maturation (144). Viruses are strongly immunogenic and the use of recombinant viral vector vaccines represents an interesting tool to generate a robust immune response by infecting APCs such as DCs, and engineering their expression of TAAs (145). Lastly, peptide vaccines are based on the identification and synthesis of antigenic epitopes derived from TAAs able to induce specific antitumor responses (146). Numerous studies have identified TAAs expressed by CRC cells as potential targets for vaccine immunotherapy, including but not limited to CEA (147, 148), WT1 (149), MUC1 (150), survivin-2B (151, 152), RNF43 (153, 154), TOMM34 (154), 5T4 (155, 156), GUCY2C (157), SART3 (158), and hTERT (159). CEA is the most extensively explored target in CRC vaccine trials and numerous phase I studies have involved CEA mRNA or CEA peptides loaded onto DCs. CEA DC vaccines were found to be safe and to induce CEA-specific T cell responses in most patients (148), and accordingly two complete responses and two cases of stable disease were observed among 12 patients (160). A study evaluated the immunogenicity of a vaccine based on MUC1 in patients without CRC cancer but with advanced colonic adenomas (precursors of CRC) and showed high levels of anti-MUC1 IgG along with long-lasting immune memory in 44% of these individuals (161). Karanikas et al. confirmed and extended these findings in CRC patients and found that 60% of vaccinated patients displayed anti-MUC1 IgG while 28% exhibited MUC1-specific CD8 T cell responses. However, this was not accompanied by a reduction of tumor size (162). Similarly, in phase I clinical trials, some patients vaccinated with HLA-A^∗^2402-restricted peptides derived from survivin-2B had stable disease but no complete responses were observed (152, 163). Recently, a phase I clinical study enrolled 10 stage I or II CRC patients who received vaccine composed of Ad5-GUCY2C-PADRE viral particles. Antibodies directed against GUCY2C- and GUCY2C-specific CD8 T cell responses were detected in 10% and 40% of patients, respectively. Although the vaccine was well tolerated, the study did not yield any clinical response in terms of tumor size reductions (164). The limited effect of these single peptide vaccines led to the development of vaccines including multiple TAA-derived peptides. In a phase I clinical study, 21 metastatic CRC patients received vaccines including both RNF43 and TOMM34 peptides in combination with chemotherapy. Interestingly, only one patient failed to mount specific CD8 T cell responses against RNF43 and/or TOMM34 and 83% of patients had stable disease following the vaccination (165). After having demonstrated the safety and immunological responsiveness of this combination therapy, the same group showed that 3-years DFS was significantly better in the group of patients with CD8 T cell responses than in the group without (154, 166). In order to improve clinical responses, Okuno et al. added to this original combination therapy 5 other TAA-derived peptides to the vaccine. This 7-peptide cocktail vaccine was able to control the disease in 60% of patients including those who had complete or partial responses and stable disease. Remarkably, the authors reported a positive association between the number of peptides against which CD8 T cells responded, and the OS. Indeed, median OS was 7 months for the 20 of 30 patients with CD8 T cell responses to 6 or less peptides, whereas it was not reached for those having CD8 T cell responses to all 7 peptides (167).

In addition to depending on TAAs, vaccine immunogenicity also relies on the use of adjuvants that are crucial components to boost and enhance antigen-specific immune responses. Most TAA-based vaccines described above are combined with different adjuvants inducing the activation and recruitment of APCs (168), such as incomplete Freund’s adjuvant (IFA) (165–167), TLR-3 agonist (161) or Flt3 ligand (160). The use of type I IFN as a second adjuvant in a survivin-2B-based vaccine already combined with IFA led to 50% of patients having stable disease versus 20% with IFA only (152). Such results highlight the critical role played by vaccine adjuvants on vaccine efficacy. However, most of these clinical studies targeting TAAs with vaccines have met with limited success with a poor benefit for CRC patients despite the use of adjuvants. These disappointing results may be at least partly due to a lack of specificity of TAAs. Indeed, although TAAs are over-expressed on tumor cells, they are often also expressed at low levels on normal cells and are consequently subject to central tolerance. T cells with high-affinity TCRs for TAAs are deleted in the thymus during negative selection to avoid autoimmunity (169). Thus, T cells having successfully undergone central tolerance process display TCRs with a lower affinity for TAAs than for foreign antigens or tumor-specific neoantigens (170). Because the intensity of T cell cytotoxicity and activation is positively correlated with TCR binding affinity, TAA-specific T cells may be less likely to elicit an effective antitumor response than neoantigen-specific T cells (171).

Recent observations that neoantigen load is associated with the clinical response to ICI in patients with cancer, along with their ability to drive potent antitumor responses have inspired the development of personalized vaccines based on tumor-specific neoantigens. The concept of this clinical approach is similar to vaccines targeting TAAs but relies on neoantigen identification and prediction using bioinformatic methods that have been reviewed by others (172). The generation of personalized neoantigen-based vaccines requires time, particularly due to the prediction and prioritization of neoantigens, but these techniques will improve and become faster and cheaper with technological advances. The efficacy of neoantigen vaccines has been demonstrated in different preclinical mouse tumor models including the CT26 and MC-38 murine colon carcinoma models by inhibiting tumor growth and eliciting efficient antitumor T cell responses (173, 174). Hence, there is increasingly a focus on defining the exact neoantigens expressed by each patient’s tumor and vaccinating against these. Thus far, this approach which is beginning to be applied to melanoma and glioma, has not been explored extensively in CRC. Neoantigen-based vaccines showed promising results in terms of neoantigen-specific CD4 and CD8 T cell responses and survival in both melanoma (175, 176) and glioblastoma (177, 178), suggesting a potential benefit in other cancers including CRC. Contrary to melanoma, a cancer with high number of mutations, glioblastoma is characterized by a low mutational load giving rise to far fewer neoantigens (80, 179). By placing these studies in the context of CRC, they suggest that a personalized neoantigen vaccine strategy might induce neoantigen-specific T cell responses and lead to clinical responses not only for patients with MSI and POLE-mutated tumors, but also for some patients harboring tumors with an MSS phenotype for whom the use of ICI alone is not a treatment option. However, as discussed above, responses to neoantigen vaccines represent only one of the multiple factors involved in the mediation of an effective anti-tumor response. Issues such as APC deficits, impaired trafficking or impaired infiltration into tumors might still prevent immune responses to neoantigens and their subsequent anti-tumor effects (180, 181). Several clinical trials with different vaccination technologies targeting neoantigens are ongoing to assess their efficiency in CRC: a personalized synthetic neoantigen vaccine in combination with an adjuvant QS-21 (NCT02992977), an mRNA-based individualized vaccine targeting tumor-associated peptides specifically expressed by the patient’s tumor cells (NCT03289962) and a frameshift-derived neoantigen-loaded DC vaccine (NCT01885702). These trials will document the utility of such approaches.

## Adoptive T-Cell Therapy in CRC

Adoptive T-cell therapy is a type of immunotherapy whereby immune cells [that can be T cells, DCs, NK cells, or cytokine-induced killer (CIK) cells] are transferred to the patient. The ability of T cells to specifically recognize tumor antigens and induce antitumor responses makes them ideal vectors for ACT. Adoptive T cell therapy consisting of harvesting, activating and expanding autologous T cells *ex vivo* before transferring them back to patients has been ongoing in other tumor types for some years (182, 183). In CRC, an early ACT clinical study with TILs involved 14 patients with liver metastases. Patients received TILs that had been extracted from these metastases, stimulated and expanded with high-dose IL-2. However, no significant difference in DFS was observed between patients treated with these TILs versus conventional chemotherapy (184). Another ACT trial involving sentinel lymph node (SLN)-T cells instead of TILs was conducted in 16 and 55 CRC patients included in a pilot study and a phase I/II clinical trial, respectively. Encouragingly, 4 complete responses, 1 partial response and 4 cases of stable disease were observed in the 9 patients with stage IV CRC in each study (185, 186). Other studies in CRC are currently ongoing (NCT03935893, NCT01174121, and NCT03904537) to confirm these preliminary results which suggest that ACT with TILs or SLN-T cells could benefit CRC patients, but also highlight a need for improvement of these therapies. Interestingly, Tran et al. isolated CD8 T cells specific for mutant KRAS G12D from TILs obtained from lung metastases of a CRC patient. The expansion and reinfusion of KRAS G12D-specific CD8 T cells into the patient led to complete regression of 6 of 7 lung metastases, but progression of the 7th. The analysis of resected tumor cells revealed a loss of expression of HLA class-I as the mechanism responsible for the latter (187).

In order to further improve the efficacy of ACT, genetic modifications of T cells to express an artificial TCR with high avidity can be achieved through gene transduction (175). In a phase I clinical trial, 3 stage IV CRC patients received autologous T cells genetically engineered to express high avidity CEA-specific TCR. A decrease in serum CEA levels was observed in all 3 patients after treatment and one patient experienced a partial response. However, the treatment led to severe toxicity as all patients developed serious inflammatory colitis (188).

CEA is also an attractive target for chimeric antigen receptors (CARs) in CRC. CARs combine an extracellular single-chain antibody variable fragment that is an antigen-binding domain and an intracellular signaling domain with co-stimulatory molecules and a T cell activating signaling domain-CD3ζ chain. CAR-T cells are T cells genetically engineered to express a receptor allowing TAA recognition through antibody binding and T cell activation thanks to the intracellular domain (176). In the last decade, CAR-T cell therapies have met with some success in hematological malignancies and efforts are currently ongoing to expand these to solid tumors (189). A phase I study reported that 7 of 10 CRC patients with CEA^+^ metastases had stable disease after transfusion of CEA-targeted CAR-T cells. Among them, 2 patients experienced tumor shrinkage and most patients showed a decrease in serum CEA levels (190). In this study, ACT with CAR-T cells was well tolerated, which unfortunately is not always the case. Indeed, a patient with metastatic CRC treated with Her-2-targeted CAR-T cells died 5 days following the infusion, probably as a consequence of cytokine release syndrome (191).

The efficiency of ACT with CAR-T cells in CRC still needs to be validated in further studies, along with optimization of the design such as the choice of TAAs or dose of CAR-T cells. Several CAR-T cells targeting different TAAs have been tested in metastatic CRC patients: EGFR (NCT03152435, NCT03542799), NKG2D, and NKG2D-ligands (NCT03370198, NCT03310008, and NCT03692429), CEA (NCT02959151, NCT03682744, and NCT02850836), C-met (NCT03638206) or EpCAM (NCT03013712). So far, these studies recruit CRC patients irrespective of their microsatellite status and further investigations will be required to assess whether CAR-T cell therapies might benefit both MSI and MSS patients.

## Conclusion

A growing body of evidence has emerged over the last few years supporting the major role played by infiltrating immune cells in tumor control and as a powerful prognostic factor in CRC. Recently, Pagès et al. included 1578 CRC patients in a combined analysis of Immunoscore and microsatellite status. This revealed that patients with high Immunoscore and MSI and patients with high Immunoscore and MSS had similar 5-year DFS rates of 75 and 72%, respectively. Additionally, no survival advantage was found for patients with MSI tumors having a low Immunoscore as compared to patients with MSS tumors (5-year DFS rates of 56 and 53%, respectively). Similar to DFS, time to recurrence and OS were prolonged in patients with a high Immunoscore, irrespective of their microsatellite status (61). This study highlights the importance of generating immune responses within immune-deserted or -excluded tumors classified into the three CMS2-4 subtypes. Immunotherapy strategies based on vaccination and particularly neoantigen vaccines might improve tumor infiltration by immune cells. Indeed, for example, the analysis of immune composition within the tumor post-vaccination, subsequent to relapse (of glioblastoma) showed an increased infiltration of CD4 and CD8 T cells comprising neoepitope-specific T cells, for the two patients who had previously responded to the vaccine, as compared to tumor at baseline (178). A deeper analysis of these infiltrating T cells revealed the expression of several inhibitory checkpoint receptors such as PD-1, TIGIT or TIM3, consistent with an exhausted phenotype (178). These observations provide a strong rationale to combine neoantigen vaccines and ICI in cancer treatment, probably also with chemotherapy or other immune modulatory (anti-suppressive) therapies. Thus, combining neoantigen vaccines with other immunotherapeutic strategies would allow treating patients without delay. For CRC patients with an MSS phenotype, a likely strategy consists of starting the treatment with vaccines targeting shared TAAs followed by neoantigen vaccines combined with ICI. In patients with MSI tumors treated with ICI, a subsequent vaccination based on neoantigens might enhance the amount of effector T cells within the tumor and reinforce the response to checkpoint blockade. Promising results have been obtained in murine melanoma and colon models where the combination of neoantigen vaccines with anti-PD-1 mAb resulted in tumor growth delay and even tumor eradication (192). Currently, this therapeutic approach is being tested in two clinical trials involving neoantigen vaccines and pembrolizumab (NCT02600949) or atezolizumab (anti-PD-L1) (NCT03289962).

Now more than ever, it is clear that environmental factors influence response to cancer immunotherapy. Recently, an unexpected link between the gut microbiota and clinically relevant antitumor responses to ICI has been identified in melanoma, RCC and NSCLC. Indeed, three independent groups showed that gut microbiota diversity and composition differ in patients responding versus patients who do not respond to anti-PD-1 mAbs (193–195). Thus, different commensals associated with efficacy of PD-1 blockade were identified as Faecalibacterium spp. or Bifidobacterium spp. in melanoma and Akkermansia muciniphila in RCC and NSCLC (193–195). As ICI treatments evolve, the gut microbiota will certainly need to be taken into account, particularly in CRC due to its close association with the intestinal microbiome.

Tumor complexity and heterogeneity in CRC and the ability of tumor cells to escape from immune surveillance by multifarious means requires a personalized treatment targeting several targets and pathways, and overcoming tumor escape mechanisms, to guarantee a more successful clinical outcome for every patient in future.

## Author Contributions

EP conceived and wrote the manuscript, and prepared the figures. GP conceived and reviewed the manuscript. EP, CV, GM, and GP contributed to revisions of the manuscript and approved it for publication.

## Conflict of Interest

The authors declare that the research was conducted in the absence of any commercial or financial relationships that could be construed as a potential conflict of interest.
